# Assessing heating distribution by therapeutic ultrasound on bone phantoms and in vitro human samples using infrared thermography

**DOI:** 10.1186/s40349-016-0058-7

**Published:** 2016-04-05

**Authors:** Gabriella Sellani, Dalila Fernandes, Abigail Nahari, Melissa Fabrício de Oliveira, Christiana Valois, Wagner C. A. Pereira, Christiano B. Machado

**Affiliations:** Biomedical Ultrasound Laboratory (Applied Research Department), Estácio de Sá University, Rua do Bispo, n. 83 - Block F, Rio Comprido, Rio de Janeiro 20261-063 Brazil; Globus Sports and Health Technology, Via Vittorio Veneto, 52, 31013 Codognè, TV Itália; Biomedical Engineering Program, COPPE/Federal University of Rio de Janeiro, Av. Horácio Macedo, 2030. Technology Center, Block H - Room H327, Cidade Universitária, Rio de Janeiro, 21941-914 Brazil

**Keywords:** Therapeutic ultrasound, Tissue-mimicking phantoms, Heating, Bioeffects, In vitro methods, Bone

## Abstract

**Background:**

Bioheat models have been proposed to predict heat distribution in multilayered biological tissues after therapeutic ultrasound (TUS) stimulation. However, evidence on its therapeutic benefit is still controversial for many clinical conditions. The aim of this study was to evaluate and to compare the TUS heating distribution on commercially available bone phantoms and in vitro femur and tibia human samples, at 1 MHz and several ultrasonic pulse regimens, by means of a thermographic image processing technique.

**Methods:**

An infrared camera was used to capture an image after each 5-min 1-MHz TUS stimulation on bone phantoms, as well as in vitro femur and tibia samples (*N* = 10). An intensity-based processing algorithm was applied to estimate temperature distribution. Sections of five femurs in the coronal plane were also used for the evaluation of heat distribution inside the medullar canal.

**Results:**

Temperature increased up to 8.2 and 9.8 °C for the femur and tibia, respectively. Moreover, the temperature increased up to 10.8 °C inside the medullar canal. Although temperature distributions inside the region of interest (ROI) were significantly different (*p* < 0.001), the average and standard deviation values for bone phantoms were more similar to the femur than to the tibia samples. Pulsed regimens caused lower increments in temperature than continuous sonication, as expected.

**Conclusions:**

Commercially available bone phantoms could be used in research focusing on thermal effects of ultrasound. Small differences in mean and standard deviation temperatures were observed between bone samples and phantoms. Temperature can reach more than 10 °C inside the medullar canal on a fixed probe position which may lead to severe cellular damage.

## Background

Therapeutic ultrasound (TUS) units are present in most of physical therapy departments worldwide, mainly for the management of musculoskeletal disorders, providing mechanical stimulation and/or heating (non-thermal and thermal effects, respectively). Among the bioeffects already evidenced are increased extensibility of collagen-rich scar tissues, tendons and joints, relief of pain and muscular spasm, stimulation of cells by upregulation of signaling molecules, activation of immune cells, remodeling of scars, and accelerating bone fracture healing [1, 2].

Several clinical trials have already shown different levels of evidence regarding the therapeutic benefit of TUS. More recently, a randomized controlled trial developed by Ilter et al. [3] showed that continuous ultrasound therapy was superior than pulsed and sham ultrasound (*p* < 0.05) in reducing resting pain scores at 6 and 12 weeks after treatment for myofascial pain syndrome patients. For subjects after whiplash injury in acute and subacute phases, Ruiz-Molinero et al. [4] obtained significant improvements in pain relief and joint mobility with TUS compared to a placebo group. On the other hand, during a systematic review using CENTRAL, MEDLINE, EMBASE, PEDro, and PsycLIT databases, Ebadi et al. [5] did not find high-quality evidence to support the use of TUS for improving pain relief or quality of life in patients with non-specific chronic low-back pain (LBP), only for improving low-back function in the short term, which was judged to be clinically non-relevant.

Acoustic transducers are of utmost importance for ultrasonic applications. They can be used to generate and receive acoustic waves through a property called piezoelectricity in which certain solid materials become electrically polarized when submitted to mechanical stress and vice versa [6]. When electrically excited, the probe generates an acoustic field which is not homogeneous. For planar transducers (commonly used by physical therapists), two regions can be distinguished: the near field (Fresnel zone) and the far field (Fraunhofer zone). In the first one, the intensity presents sharp variations, while in the other, it smoothly decreases with distance from the transducer. For physical therapy practice, one may be concerned that the near field is commonly used for therapeutics, and its inhomogeneous acoustic field may lead to high- and low-intensity regions by wave constructive and destructive interference, respectively [7]. Moreover, a technical report published by Artho et al. [8] has shown that the intensity displayed on TUS units does not always correspond to the actual emitted output, raising questions about safety in physical therapy daily practice.

Thermal effect of ultrasound is mainly due to a phenomenon called absorption, in which the mechanical energy is converted into heat. Heating is related to the distribution of intensity in the absorbed beam and the frequency-dependent absorption coefficient (characteristic of each type of tissue). However, the transducer itself can be a source of heat by conduction (direct contact with the skin), and one should also consider blood perfusion which plays a key role in reducing heating significantly. The temperature rise in the body is always a matter of great concern because of its influence in cellular activity [9].

Bioheat models have already been proposed to predict acoustic heat distribution in multilayered biological tissues [10–12]. However, there are still some questions regarding dosage, wave emission parameters, etc. Besides, the clinical use of TUS in physical therapy has been justified on alleged physiological responses to its biophysical effects. Consequently, clinical protocols have been adopted even in the absence of clinical studies [13, 14].

In light of this situation, there is a need to continuously propose techniques to assess the effects produced by ultrasound in the human body with higher confidence and accuracy. Tissue-mimicking phantom materials have been largely explored in ultrasound research in the last decades [15–19]. They represent an interesting alternative to in vitro samples or even to patient exposure, since several parameters (size, mechanical and thermal properties, etc.) can be easily controlled, and they can be reused many times with no ethical issues [20, 21]. Concerning bone, commercially available phantoms (Sawbones®, USA) have been used in several studies on ultrasound propagation in bone [22, 23] to estimate quantitative parameters (speed of sound, backscattering, and attenuation coefficient); nevertheless, it is not known whether they can be used for thermal phenomena studies. Lioce et al. [24] have recently assessed thermal and mechanical effects of TUS on a joint-mimicking phantom using a muscle-equivalent material. Although some results are interesting, temperature was measured locally (using thermal probes). Sellani et al. [25] showed preliminary results in comparing the heating distribution from thermographic images in a block and cylindrical cortical bone phantom and in vitro human femur samples using a 1-MHz TUS stimulation, indicating that these phantoms could be used for studies on ultrasonic thermal effects.

Recognizing that further research is needed to better clarify the bioeffects of ultrasound and that phantom materials have been proposed to substitute biological tissues in some scientific approaches, this technical report moves forward and presents an evaluation and comparison of the therapeutic ultrasound heating distribution on commercially available bone phantoms and in vitro femur and tibia human samples, at 1 MHz and several ultrasonic pulse regimens, by means of a thermographic image processing technique. More specifically, this work intends to (1) compare the heating distributions among the surfaces of a block and cylindrical cortical bone phantom and in vitro samples of human femur and tibia; (2) evaluate the heating distribution inside the medullar canal during TUS cortical stimulation; and (3) present a methodology for thermographic image processing to extract information on heating distributions after TUS stimulation. Although the method presented here does not intend to simulate realistic clinical conditions, it may provide insight into possible effects of heating by TUS protocols.

## Methods

### Samples

**Fig. 1:**
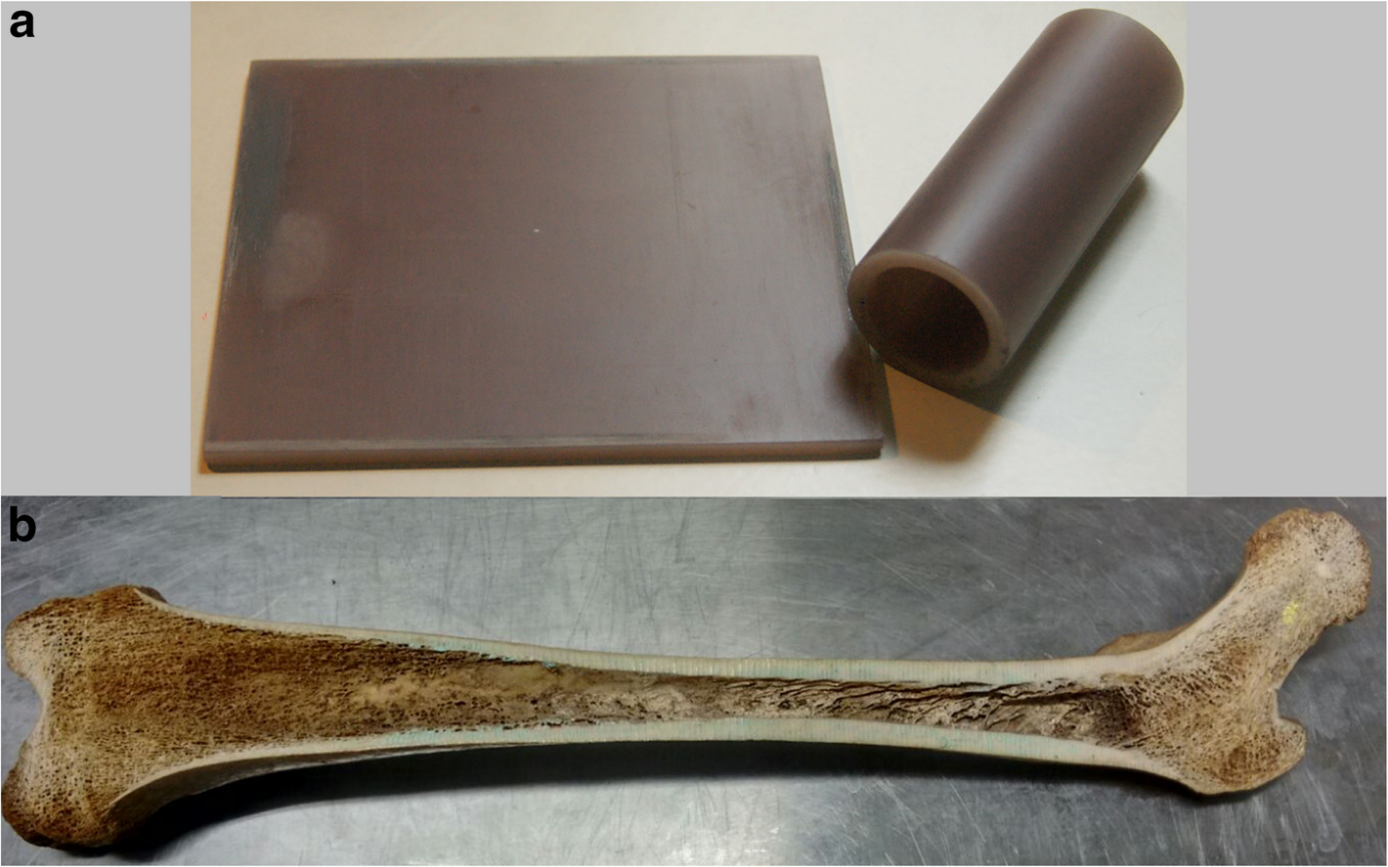
Images of the samples used in this study. **a** Commercially available cortical bone-mimicking phantoms (Sawbones®, USA): a 4-mm-thick short fiber-filled epoxy block and a 5-mm-thick 40-mm-diameter cylinder. (**b**) One of the in vitro human femurs cut in the coronal plane with a saw

Two commercially available cortical bone-mimicking phantoms were used (Sawbones®, USA): a 4-mm-thick short fiber-filled epoxy block (Ph_bl_) and a 5-mm-thick 40-mm-diameter cylinder (Ph_cyl_) (Fig. 1a). These phantoms simulate the mechanical properties of the cortical bone [26]. A total of fifteen in vitro human bone samples from an Anatomy Laboratory were studied: five intact femurs (F1 to F5—total length = 43.58 cm ± 1.47 cm; 1/2 diaphysis side-to-side diameter = 2.68 cm ± 0.18 cm), five intact tibias (T1 to T5—total length = 34.94 cm ± 1.29 cm; 1/2 diaphysis side-to-side diameter = 3.14 cm ± 0.27 cm), and other five in vitro femurs cut in the coronal plane with a saw (Fc1 to Fc5—total length = 42.52 cm ± 1.70 cm; 1/2 diaphysis side-to-side diameter = 2.38 cm ± 0.07 cm) for the analysis of heating distribution inside the medullar canal, as shown in Fig. 1b. Soft tissue was completely removed from all bone specimens. Some mechanical, acoustic and thermal properties of the phantom and cortical bone are presented in Table 1.

**Table 1:** Some mechanic, acoustic, and thermal properties of Sawbones® phantoms and cortical bone

Physical property	Sawbones®	Cortical bone
Density (kg/m^3^)	1700^a^	1810^b^
Tensile strength (ultimate) (MPa)	90^a^	124^b^
Modulus of elasticity (GPa)	12.4^a^	17.6^b^
Acoustic longitudinal velocity (m/s)	3300^c^	4200^c^
Acoustic transverse velocity (m/s)	1600^c^	2000^c^
Acoustic attenuation (dB/cm MHz)	5.7–6.2^d^	1–10^e^
Specific heat (J/kg °C)	1.25^d^	1.25^f^
Thermal conductivity (W/m K)	0.47^d^	0.31^f^

^a^Information provided by the manufacturer

^b^Ref [36]

^c^Ref [22]

^d^Measured by our laboratory

^e^Ref [37]

^f^Ref [29, 30]

This work was previously approved by the Estácio de Sá University Ethics Committee, Rio de Janeiro, Brazil, in October 10, 2013 (protocol number 20199113.4.0000.5284), and it was performed in accordance with the Declaration of Helsinki. The present study will also use preliminary reports published by the same group [25] for a comparison analysis.

### Experimental setup (surface heating)

**Fig. 2:**
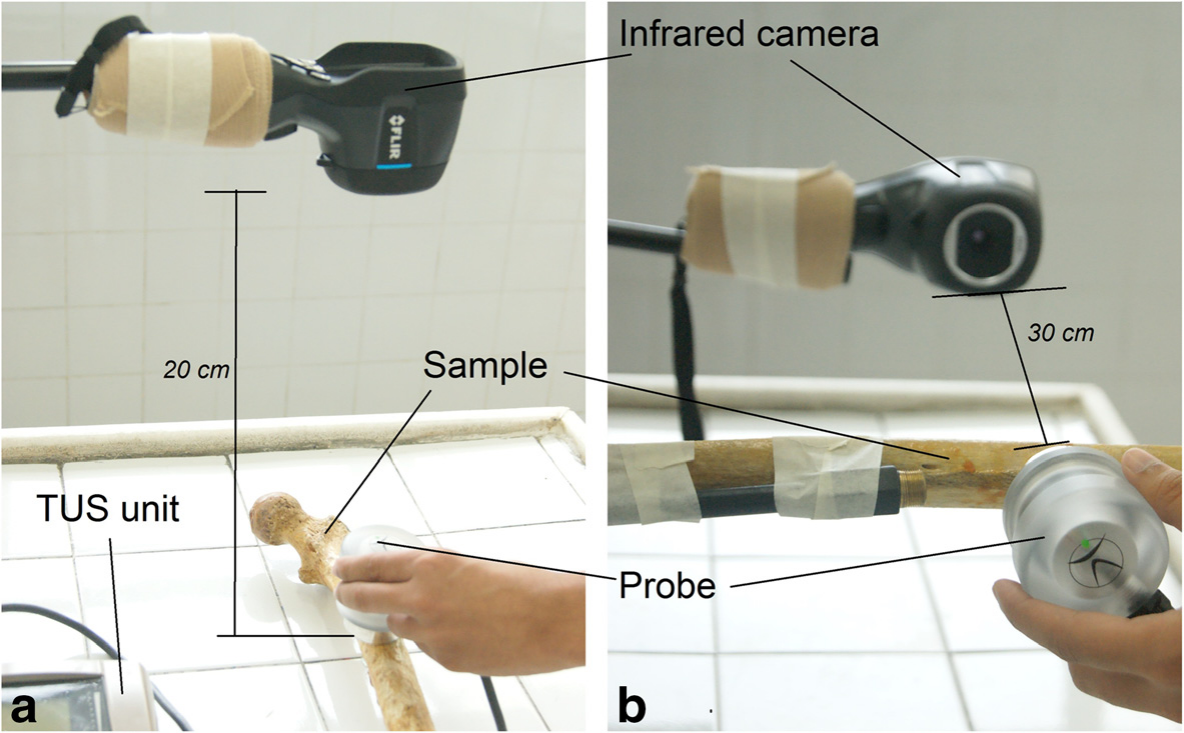
Experimental setups used in this study. **a** Experimental setup of the “surface heating” protocol showing the position of the infrared camera and TUS unit. Probe is above the sample (phantom or bone specimen). **b** Experimental setup of the “medullar canal” protocol. The infrared camera was positioned at 30 cm from the internal surface of the sample. The TUS probe was placed in contact with the sample on its external surface using coupling gel

The TUS equipment was a LipoZero Bellissima 100 (Globus®, Italy) at 1 MHz, with a nominal SATA (spatial average-temporal average) intensity of 1 W/cm^2^ and a probe head with a diameter of 4.2 cm. Three pulsed regimens were studied: continuous, pulsed 1:2 (0.5-s on, 0.5-s off), and pulsed 1:10 (0.1-s on, 0.9-s off). As shown in Fig. 2a, the TUS probe was placed in contact with the phantom (Ph_bl_ and Ph_cyl_) or bone specimens F1–F5 and T1–T5 with coupling gel and was not moved during the entire process. The unit stimulated each sample/pulsed regimen during 5 min. Temperature (in °C) on sample surface was constantly computed before simulation (at *T*_0_) using thermography. A heated tape with a length of 5 cm was stuck on the sample for distance calibration purposes during image processing.

A thermographic infrared camera was used to capture the heating distribution (i7, Flir® Systems Inc., USA; thermal sensitivity: 0.1 °C; image resolution: 140 × 140; pixels spectrum: 7.5 to 13 μm; precision: ±2 °C). It was used with an emissivity of 0.99, since according to literature, it would lead to a potential error rate of 1 % of apparent surface temperatures measured in bone [27]. The camera was placed 20 cm above and parallel to the surface of each sample, and an image was captured 5 min after the beginning of the ultrasonic stimulation, with the removal of the TUS probe. Room temperature was controlled between 24.5 and 25.5 °C using a temperature meter (B&K Precision 710, USA; resolution: 0.1 °C; accuracy: ±0.2 % reading + 0.1 °C; K-type thermocouple). Images were then stored as a matrix in which each pixel represents the local temperature value (°C) for image processing.

### Experimental setup (medullar canal)

The same experimental setup described above was applied to the block phantom (referred now as Ph_bl_^*^) and samples Fc1 to Fc5. This is depicted in Fig. 2b. The infrared camera was positioned at 30 cm from the internal surface of the sample. The TUS probe was placed in contact with the sample on its external surface using coupling gel, and it was kept fixed during the stimulation. All TUS parameters (pulsed regimen, frequency, time of stimulation, image acquisition protocol) were the same.

### Image processing and statistical analysis

**Fig. 3:**
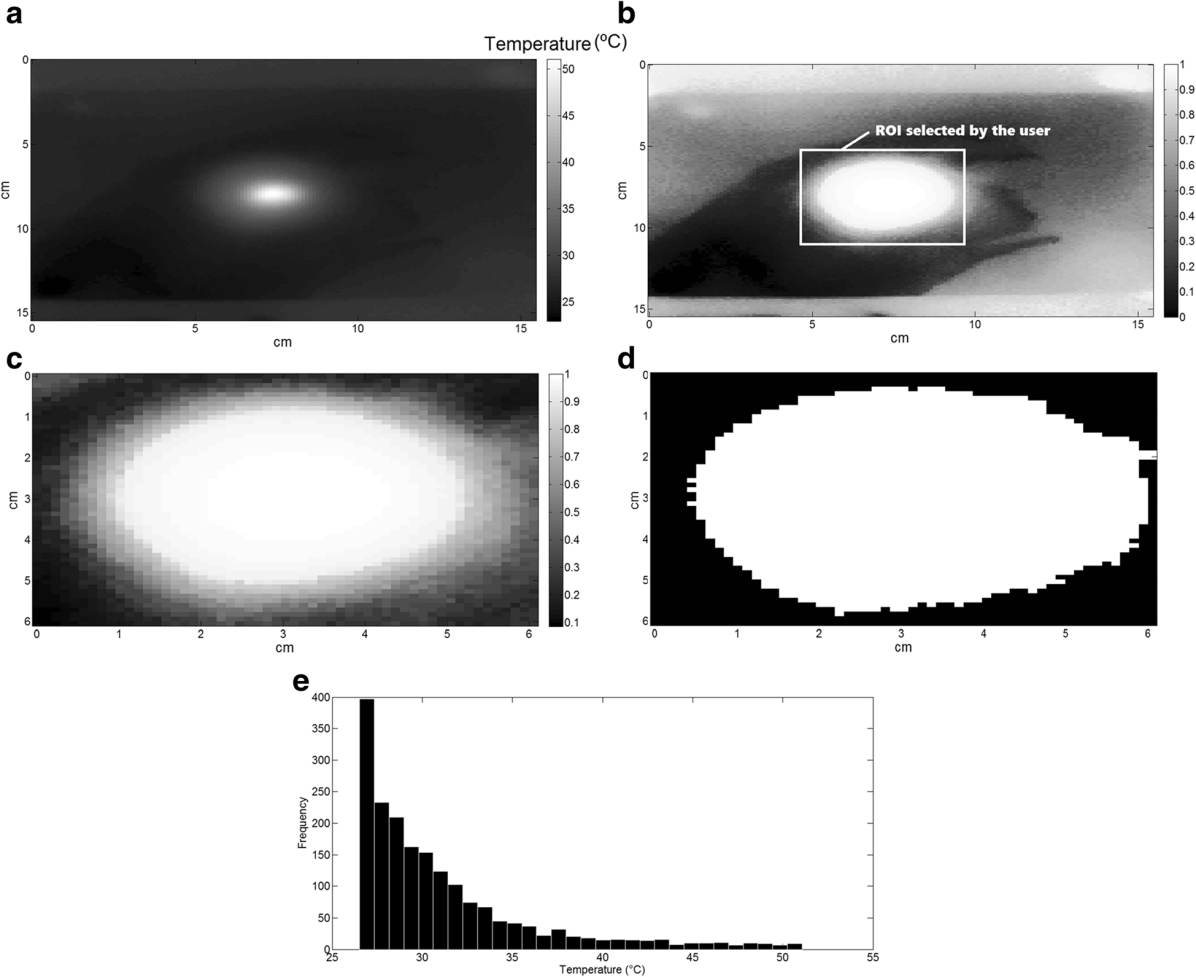
Image processing algorithm. **a** The original image is obtained as a matrix with 140 × 140 pixels, and each pixel is represented by a temperature value, as captured by the infrared camera. **b** A contrast enhancement of the normalized gray-scale image using histogram equalization is performed, and the user is able to correctly select the ROI. **c** Zoom of the selected ROI. **d** After the determination of an intensity threshold, the algorithm separates and binarizes (turns image pixels in black and white) the heating area from the background. **e** Once the heating area is isolated, the histogram with the temperatures inside the ROI is depicted, and *X*_T_ and SD_T_ can be estimated (using the temperature data from the original image)

An algorithm for image processing was implemented in Matlab® (MathWorks Inc., USA) to develop a simple normalized gray-scale intensity-based technique [28] for the segmentation (delineation) of the heating area and to compute the mean (*X*_T_) and standard deviation (SD_T_) of temperatures (°C) inside a region of interest (ROI) with area *S*_ROI_ (given in cm^2^). The following steps were taken (Fig. 3): (1) the image with the heated tape is used to calibrate for distance (i.e., the algorithm considers that the length of the tape in the image is 5 cm); (2) image contrast enhancement using histogram equalization; (3) the user selects of a ROI (which is the heating area from the image) using the mouse; (4) the user determines a pixel intensity threshold to separate (and binarize) the heating area from the background; (5) finally *X*_*T*_, SD_T_, and *S*_ROI_ are estimated in the original ROI image, as well as the variable Δ*T* (the difference between *X*_T_ and *T*_0_).

For statistical analysis, Kolmogorov-Smirnov tests were applied to evaluate the null hypothesis that temperature distributions inside the ROI were the same between phantoms and bone samples. The level of significance was 0.05.

## Results

**Fig. 4:**
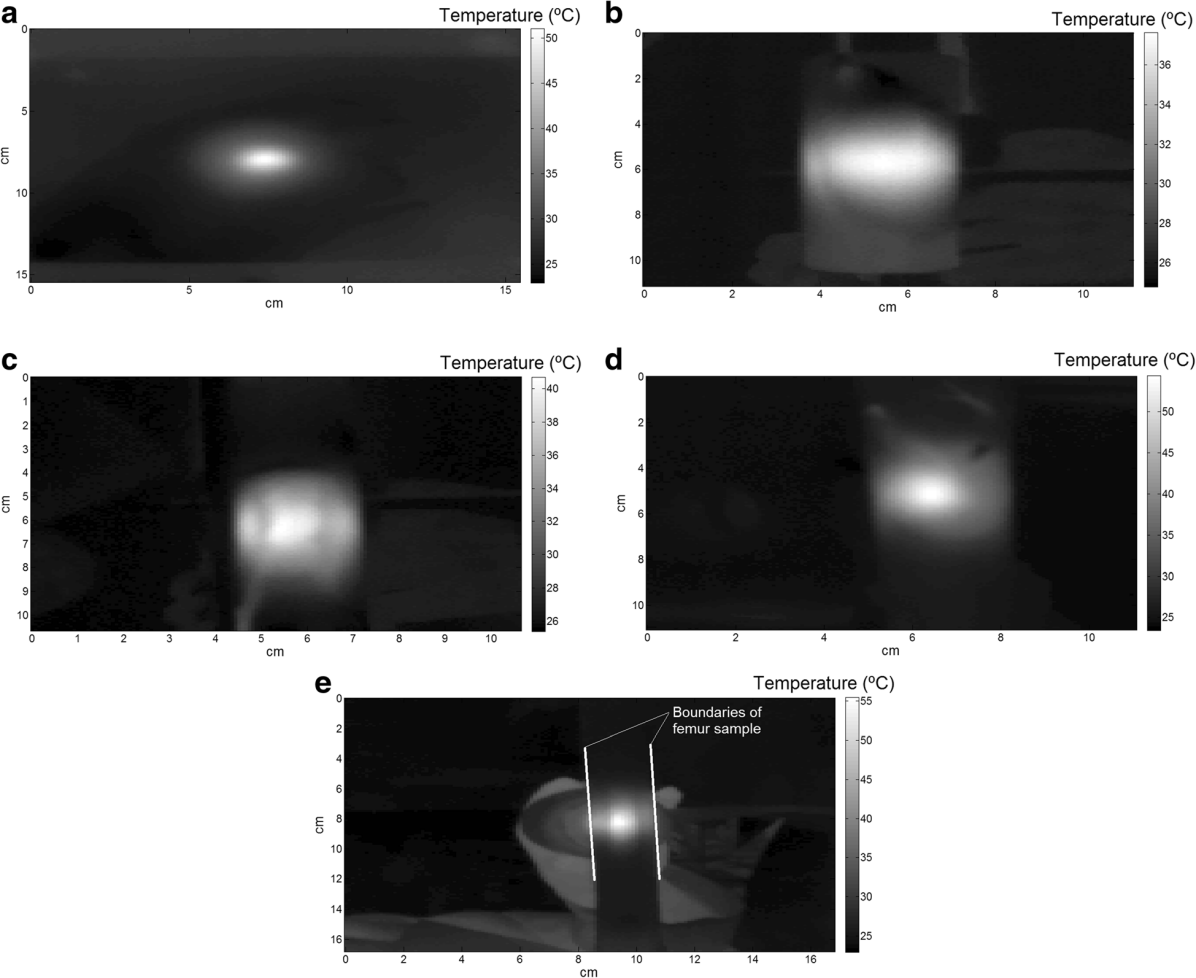
Examples of thermographic images (continuous regimen, after 5 min of stimulation). **a** Block bone phantom. **b** Cylinder bone phantom. **c** F1 sample. **d** T1 sample. **e** Fc2 sample

**Fig. 5:**
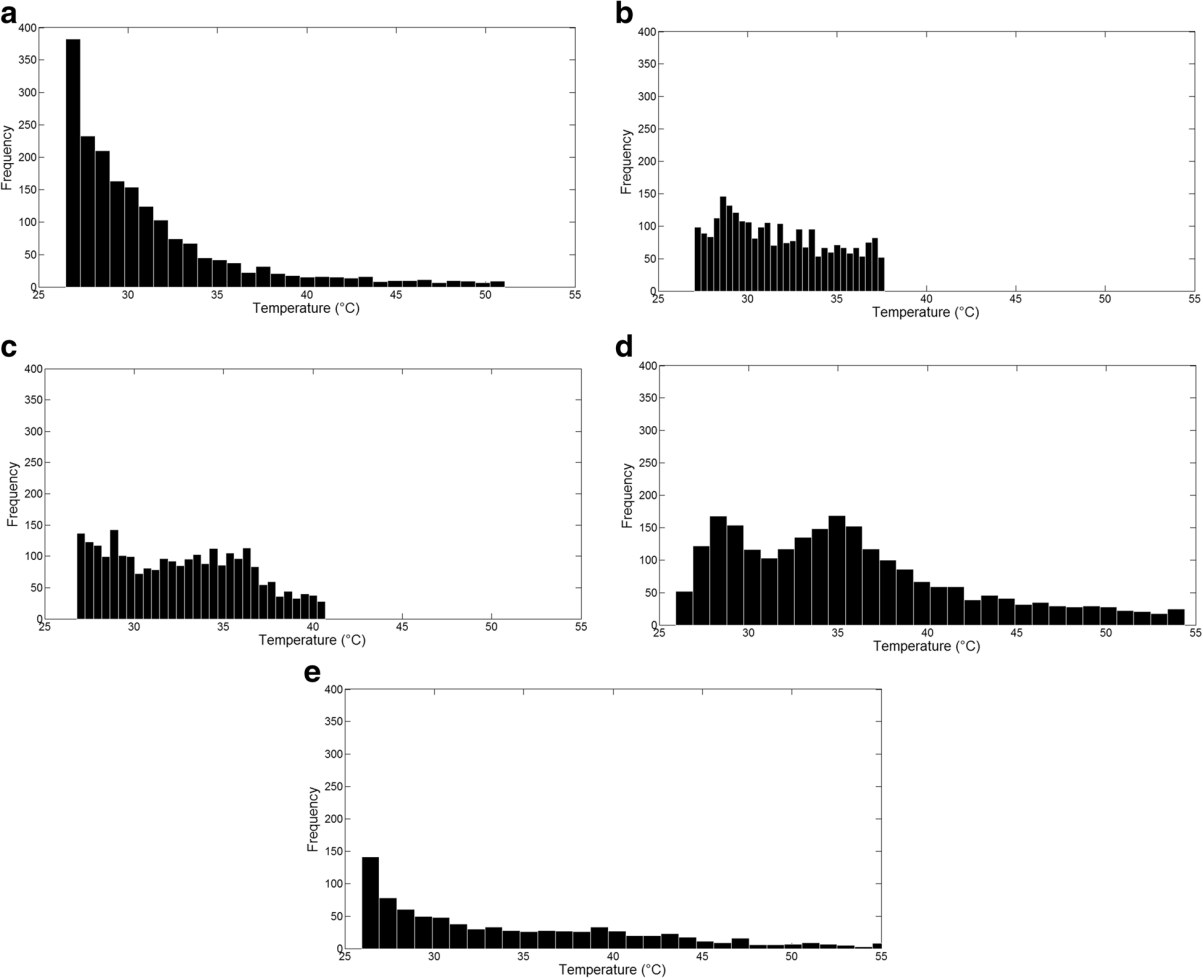
Histograms with temperatures values inside the chosen ROI from the images in Fig. 5. **a** Block bone phantom. **b** Cylinder bone phantom. **c** F1 sample. **d** T1 sample. **e** Fc2 sample

Figure 4a, d shows thermographic images from the “surface heating” setup and Fig. 4e from the “medullar canal” setup, after ultrasound stimulation with the continuous regimen, during 5 min. The first three images (a–c) were captured from the block and cylinder phantoms and femur sample F1. Figure 4d, e contains images captured from samples T1 and Fc2. It is possible to observe different temperature distributions with a visual observation. Histograms with temperature values obtained from the images in Fig. 4 are presented in Fig. 5.

Values of *X*_T_, SD_T_, Δ*T*, and heating area for all samples, pulsed regimens, and protocols (surface heating and medullar canal) are depicted in Tables 2, 3, and 4. Different results were obtained among the samples. Kolmogorov-Smirnov tests have shown that the distributions are significantly different (*p* < 0.001). When comparing continuous and pulsed regimens, the increase in temperature is higher in continuous mode, and it decreases as the number of pulses per time unit increases.

**Table 2:** Mean (*X*_T_), standard deviation (SD_T_), *ΔT*, and heating area (ROI) for each phantom and sample (continuous regimen)

Sample	*X*_T_ (°C)	SD_T_ (°C)	*ΔT* (°C)	ROI—heating area (cm^2^)
Ph_bl_	32.5	4.4	6.1	17.9
Ph_cyl_	31.7	3.0	5.4	16.3
F1	32.6	3.7	6.5	14.8
F2	33.8	4.3	7.0	12.6
F3	31.7	3.0	5.5	13.5
F4	32.1	3.7	8.2	8.3
F5	30.3	3.2	5.7	12.9
T1	35.7	6.6	9.8	13.5
T2	32.4	5.2	7.0	16.8
T3	32.1	5.2	8.4	15.8
T4	33.3	4.5	9.1	16.1
T5	32.9	5.0	8.4	18.7
Ph_bl_^*^	30.2	4.8	4.2	46.7
Fc1	35.4	8.1	10.8	7.9
Fc2	34.2	7.4	8.7	11.6
Fc3	31.5	6.1	7.7	9.4
Fc4	34.3	8.4	9.9	8.6
Fc5	34.7	7.1	8.8	11.7

**Table 3:** Mean (*X*_T_), standard deviation (SD_T_), *ΔT*, and heating area (ROI) for each phantom and sample (1:2 pulsed regimen)

Sample	*X*_T_ (°C)	SD_T_ (°C)	*ΔT* (°C)	ROI—heating area (cm^2^)
Ph_bl_	29.7	2.9	3.3	17.9
Ph_cyl_	30.2	1.9	3.4	14.5
F1	30.2	1.9	3.5	12.7
F2	30.0	2.9	4.5	14.4
F3	29.6	1.9	4.3	12.0
F4	30.1	2.4	5.8	7.0
F5	29.7	1.7	4.3	8.0
T1	30.9	3.8	6.4	18.5
T2	31.0	3.5	6.4	16.7
T3	29.2	3.1	5.9	16.8
T4	29.9	3.7	5.9	18.8
T5	29.6	3.5	5.4	14.9
Ph_bl_^*^	26.5	3.8	1.8	23.7
Fc1	29.8	4.8	5.5	11.8
Fc2	29.6	4.5	5.0	14.7
Fc3	30.5	5.1	5.7	11.3
Fc4	30.1	4.2	5.8	16.0
Fc5	30.5	5.0	5.3	13.3

**Table 4:** Mean (*X*_*T*_), standard deviation (*SD*_*T*_), *ΔT*, and heating area (ROI) for each phantom and sample (1:10 pulsed regimen)

Sample	*X*_T_ (°C)	SD_T_ (°C)	*ΔT* (°C)	ROI—heating area (cm^2^)
Ph_bl_	26.3	0.5	0.3	10.1
Ph_cyl_	26.5	0.3	0.8	8.7
F1	27.7	0.6	2.9	9.2
F2	27.1	0.5	2.1	7.2
F3	27.1	0.3	2.1	7.0
F4	26.1	0.2	2.1	2.3
F5	25.8	0.3	1.9	4.1
T1	26.8	0.7	2.6	11.8
T2	25.8	0.7	1.5	12.6
T3	26.5	0.5	2.3	10.0
T4	26.8	0.7	1.0	11.6
T5	27.2	0.5	2.0	10.5
Ph_bl_^*^	26.1	1.1	1.2	15.5
Fc1	26.3	1.1	2.2	11.7
Fc2	26.2	1.1	1.3	9.1
Fc3	26.3	1.1	1.5	6.4
Fc4	26.2	0.8	0.8	9.9
Fc5	26.2	0.8	0.8	10.3

## Discussion

This work presented original data using 1-MHz ultrasound heating in bone phantoms and in vitro bones using a processing technique for thermographic images. Results obtained for femur samples were already presented elsewhere by Sellani et al. [25]. Although temperature distributions inside the ROI were significantly different between bone phantoms and specimens, one may say that average and standard deviation values for bone phantoms were more similar to the femur (as also presented by Sellani et al. [25]) than to the tibia samples, maybe due to their similar thickness (4–5 mm). It was possible to observe that, within 5 min of stimulation, and for a continuous regimen, temperature increased up to 8.2 °C [25] and 9.8 °C for femur and tibia, respectively. Moreover, the temperature increased up to 10.8 °C inside the medullar canal (Table 2). It is known that increases of more than 6 °C lead to detrimental effects, and it is often taken as cellular damage threshold; increases up to 9 °C can cause coagulation of protein and enzymes denaturation [9]. However, this heating pattern is not common in low-intensity physiotherapeutic treatments (approximately 1–1.5 W/cm^2^) applied to body regions with a considerable amount of muscle layer, since homeostatic mechanisms tend to counteract the rise in temperature of tissues exposed to heating [14]. On the other hand, it could be hypothesized that for TUS treatments at body regions where the cortical bone is under small layers of soft tissue (for instance, the anterior aspect of tibia, posterior aspect of radius, ribs, hands and feet bones), higher temperatures could be achieved with an exposure of some minutes. This could be a subject of a new investigation in the future.

As expected, pulsed regimens (1:2 and 1:10) caused lower increments in temperature, as seen in Tables 3 and 4; however, it is important to note that higher temperature values could be achieved with a 1:2 regimen (5.8 and 6.4 °C, respectively). The different temperature distributions, as assessed with the histograms and thermographic images, may be due to macroscopic differences among human samples (e.g., the curvature in the cylinder phantom is more regular than in bone samples and cortical thickness and bone mineral density vary for each bone sample). It is worth mentioning that a repeatability analysis was performed before this experiment, for the short fiber-filled epoxy block, continuous mode [25] and low variations were obtained for *X*_T_ (±0.5 °C) and SD_T_ (±0.1 °C). The phantoms used in this work present different thermal conductivities (*k*) and similar specific heat (*c*) when compared to the cortical bone [29, 30] (*k* = 0.47 W/m K and 0.31 W/m K, respectively; *c* = 1.25 J/kg °C for both). It is known that *k* indicates the capacity of a material to conduct heat; thus, it is expected that the heat transfer in the phantom specimen would occur at a higher rate than in bone. However, this phenomenon could not be observed with the applied methodology. The similar specific heat between them may corroborate the small differences in *X*_T_ and SD_T_ values (Tables 1, 2, and 3).

This is the first time that thermography is used to evaluate thermal effects of ultrasound in in vitro human samples, giving information about heating distribution in the sample surface in two dimensions. Image processing provided average and standard deviation values from the heating area. In the study of Lioce et al. [24], a joint-mimicking phantom using a muscle-equivalent material was considered to assess TUS effects. Nevertheless, thermal probes were applied in fixed positions to acquire temperature data also during 5 min of stimulation. Increases in temperature up to 17 °C were observed using a continuous regimen. Taking into account the standard deviation values from the present study (up to 8.4 °C, Table 1), it could be said that the results are similar. Teixeira et al. [31] developed a three-layer (non-homogeneous) phantom using glycerin, agar, and graphite, for non-invasive temperature estimation during TUS stimulation by means of five thermocouples, placed along the transducers axial line, not providing the high-resolution spatial heating data as thermography does. Oliveira et al. [32] presented a cylindrical four-layer phantom: soft tissues (including fat, muscle, and bone marrow) and the cortical bone (using the same phantom of the present work) to evaluate (numerically and experimentally) the heat propagation through the layers by means of thermography. The images were taken from an inside perspective (along the layers, transverse section of the cylinder). The mean and maximum temperatures were used as parameters. Values were very close to the ones obtained here, indicating that the presence of soft tissue may not considerably vary temperature distribution on cortical surface.

All the image processing techniques (equalization, binarization) were done to correctly select the ROI. After this selection, all the estimations (average, standard deviation) were obtained from the original image, inside the selected ROI. Histogram equalization is commonly applied in image processing for contrast adjustment. It is possible to increase the gain of lower local contrasts resulting in higher contrast areas, and then spreading out the most frequent intensity values. During the development of the processing algorithm, it was observed that it was easier to select the correct ROI with contrast enhancement before ROI selection (i.e., the heated region in the sample). One may also say that the bone curved surfaces were not taken into account by the imaging device thus the effect of curvature in the analysis of temperature distributions was neglected.

Similar to other studies, some limitations of this study include the absence of blood perfusion and metabolic compensation. Also, the proposed experimental setup is far from reality (the transducer in contact with bone surface) and soft tissues are not present (although when tibia is considered, one can say that the experimental setup is closer to reality—the anterior surface of tibia is separated from the outside only by skin and other slim membranes of connective tissue) [33]. According to Valvano [34], temperature and water content play a key role on thermal properties, and in vivo heat transfer is dependent on blood flow, extracelullar water, and local metabolism. Some of the temperature increase (mainly for the surface heating setup) may be due to heat conduction from transducer self-heating. Finally, ter Haar et al. [35] proposed four levels used to describe the nature and quality of the ultrasonic exposure data: the results presented here could be classified as level 1 (there is an indicator of output for a physical therapy system, but no actual acoustic output measurements made).

Alassaf et al. [21] have highlighted the importance of in vitro tests, since as investigators continue to propose and develop novel applications of TUS, there is a need to offer a diverse range of information as well as to reduce the demand on animal testing. Tissue-mimicking phantoms have been largely used in applied research as an interesting alternative: there is the possibility of controlling several parameters like size and mechanical and thermal properties, without any ethical issue. Specifically in the case of Sawbones® phantoms, they were designed to mimic the mechanical properties of the bone, but some investigators have presented studies on the ultrasound propagation in this material. To the best of our knowledge, no study used these phantoms to analyze thermal phenomena from ultrasound stimulation.

We are aware that reflection and refraction phenomena occur in the propagation path of the ultrasonic wave as it passes through skin, fat, and muscles. In this work the probe was put in contact to bone surface. On the other hand, literature shows that the acoustic intensity arrives at the cortical surface with small losses. Lin et al. [11] have shown that the maximum ultrasound energy absorption is always at the interface of muscle and bone, and the temperature peak is located beyond the cortical bone interface. If a muscle has a low-perfusion profile or a bone presents a higher acoustic attenuation, the maximum temperature shifts closer to the interface.

Finally, three contributions can be highlighted in this work: (a) it provided information about thermal responses from commercially available bone phantoms, which are already used for ultrasound non-destructive tests, comparing them with in vitro human samples; (b) it showed that the temperature distribution on the surface of bone samples after TUS stimulation, and the possibility to use image processing techniques to better extract information about heating pattern; and (c) it was possible to evaluate the heating distribution inside the medullar canal, mainly in situations where the physical therapists are applying ultrasound in body sites with bones near the skin. The effect of blood flow and metabolic compensation are still questions to be answered. Future research includes the use of soft tissue phantoms and in vitro animal samples over bone in the experimental model, as well as data collection with the samples immersed on a temperature-controlled water bath to simulate more physiological conditions. Imaging of bone samples (microtomography) to estimate cortical thickness in an attempt to correlate with heat transfer through the cortical shell will also be an interesting approach.

## Conclusions

The findings of this work show that commercially available bone phantoms could be used in research focusing on thermal effects of ultrasound. Considering a 1-MHz pulse and 5 min of TUS stimulation (continuous and pulsed regimens), small (but significant) differences in temperature distribution were observed between the femur and tibia samples and phantoms, by means of an intensity-based processing technique for thermographic images. Bone type, cortical thickness, and bone geometry may have caused different temperature distributions among the samples. On the other hand, if TUS stimulation is done on a fixed probe position, temperature increases can reach more than 10 °C inside the medullar canal, causing severe cellular damage.

This in vitro approach is far from clinical reality due to the limitations previously exposed. More realistic protocols (for instance, using implanted thermocouples) would be necessary to assess in vivo TUS effects in animal models and humans (of course, following the ethical issues). Questions have to be pointed out in the future, to better understand the underlying mechanisms of ultrasound propagation and their effect in biological tissues.
